# Weight Loss by Ppc-1, a Novel Small Molecule Mitochondrial Uncoupler Derived from Slime Mold

**DOI:** 10.1371/journal.pone.0117088

**Published:** 2015-02-10

**Authors:** Toshiyuki Suzuki, Haruhisa Kikuchi, Masato Ogura, Miwako K. Homma, Yoshiteru Oshima, Yoshimi Homma

**Affiliations:** 1 Fukushima Medical University School of Medicine, Fukushima, 960–1295, Japan; 2 Graduate School of Pharmaceutical Sciences, Tohoku University, Sendai, 980–8678, Japan; University of Pécs Medical School, HUNGARY

## Abstract

Mitochondria play a key role in diverse processes including ATP synthesis and apoptosis. Mitochondrial function can be studied using inhibitors of respiration, and new agents are valuable for discovering novel mechanisms involved in mitochondrial regulation. Here, we screened small molecules derived from slime molds and other microorganisms for their effects on mitochondrial oxygen consumption. We identified Ppc-1 as a novel molecule which stimulates oxygen consumption without adverse effects on ATP production. The kinetic behavior of Ppc-1 suggests its function as a mitochondrial uncoupler. Serial administration of Ppc-1 into mice suppressed weight gain with no abnormal effects on liver or kidney tissues, and no evidence of tumor formation. Serum fatty acid levels were significantly elevated in mice treated with Ppc-1, while body fat content remained low. After a single administration, Ppc-1 distributes into various tissues of individual animals at low levels. Ppc-1 stimulates adipocytes in culture to release fatty acids, which might explain the elevated serum fatty acids in Ppc-1-treated mice. The results suggest that Ppc-1 is a unique mitochondrial regulator which will be a valuable tool for mitochondrial research as well as the development of new drugs to treat obesity.

## Introduction

Mitochondria are organelles comprised of an outer and inner membrane, enclosing a matrix space. Within the inner membrane is a series of redox enzymes comprising the respiratory chain, which reduces oxygen to water using electrons generated by dehydrogenases employing intermediary metabolite substrates. The respiratory enzyme complexes are redox-driven proton pumps, which translocate protons from the matrix space into the inner mitochondrial compartment, thus establishing an electrochemical potential gradient across the inner mitochondrial membrane. The electrochemical gradient is utilized by the reversible proton pumping function of ATP synthase to synthesize ATP from ADP and inorganic phosphate. Various small compounds have been used to study mitochondrial regulation [1–3]. Rotenone [4] and antimycin A [5] are well-known inhibitors of respiratory chain complex I and III, respectively, while oligomycin A is a specific inhibitor of ATP synthase. In addition, uncoupling agents such as 2,4-dinitrophenol (DNP) [6] and carbonyl cyanide m-chlorophenyl hydrazone (CCCP) [7] are useful probes for understanding the mechanisms involved in electron transfer and ATP production. DNP and CCCP are well-characterized mitochondrial uncoupling agents, and act as a protonophore to shuttle protons across the mitochondrial inner membrane, leading to dissipation of the mitochondrial proton gradient and conversion of the energy derived from mitochondrial substrate oxidation into heat.

Cellular slime molds are soil microorganisms that produce many pharmacologically active compounds, and are an important source of lead compounds for medical research. The slime mold *Dictyostelium discoideum* forms a fruiting body consisting of spores and a multicellular stalk at the end of its life cycle, in response to differentiation inducing factor-1 (DIF-1), a putative morphogen which induces stalk cell differentiation [8]. The first metabolite produced during DIF-1 degradation is DIF-3, which unlike DIF-1 has virtually no activity in the induction of stalk cell differentiation [9]. Nevertheless, both DIF-1 and DIF-3 possess anti-tumor activity, suppressing mammalian cell proliferation and, in some cases, inducing or promoting the differentiation of de-differentiated tumor cells *in vitro* [10–16]. Recently, we showed that DIF-3 and its analogues induce morphological changes and dysfunction of mitochondria, suggesting that DIF-like molecules suppress cell proliferation, at least in part, by interfering with mitochondrial activity [17,18]. In this study, we screened natural low-molecular-weight compounds derived from *D*. *discoideum* and other microorganisms to identify novel mitochondrial modulators. We report that the secondary metabolite, Ppc-1, is a unique compound which enhances mitochondrial oxygen consumption and induces weight loss in mice. Thus, Ppc-1 is novel and potentially valuable small molecule reagent for mitochondrial research.

## Results

### Oxygen consumption of natural compounds

**Figure 1:**
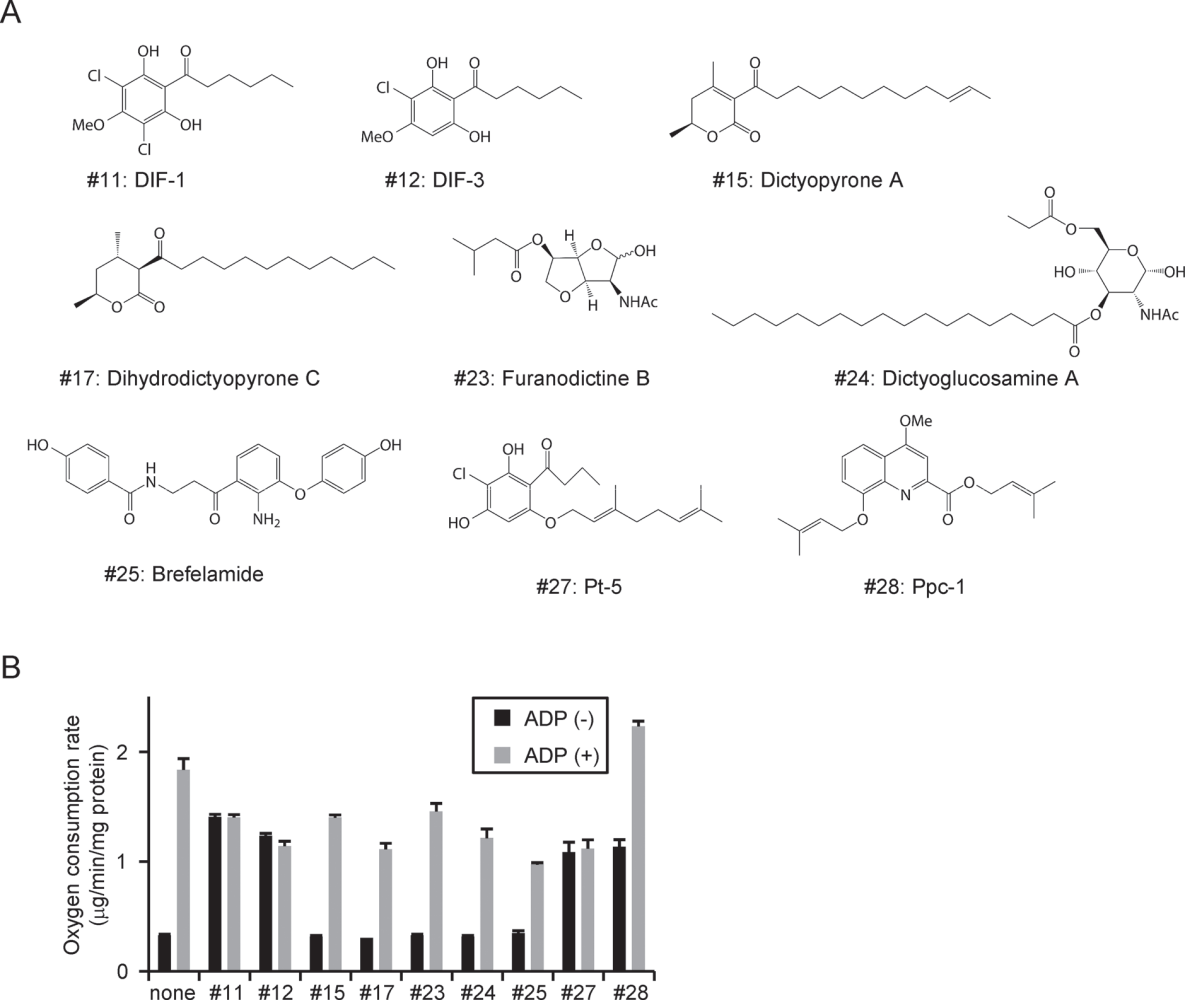
Screening of uncoupling agents. (A) Structures of small molecule compounds identified in slime molds. (B) Effects of compounds (20 μM each) on oxygen consumption were measured in the absence or presence (state 3) of ADP using isolated mitochondria. Four compounds were found to enhance oxygen consumption, suggesting uncoupling activity. Only Ppc-1 exhibited no inhibitory effect on ‘state 3’.

We screened for novel modulators of mitochondrial function by measuring oxygen consumption rates in mitochondria-enriched fractions prepared from mouse liver. We tested 32 natural small molecule compounds, including 9 derived from slime molds (Fig. 1A) [8,9,19–24]. Assays were carried out in buffer containing 100 μM palmitoyl-carnitine as a substrate for oxidation, and monitoring oxygen levels using an oxygen electrode. After preincubating the mitochondrial-enriched fraction in an assay buffer at 30°C for 30 s, each test compound was added to a final concentration of 20 μM, followed by addition of ADP to a final concentration of 400 μM, in order to induce ‘state 3’. Four of the nine compounds derived from slime molds enhanced the oxygen consumption rate in the absence of ADP, including #11, #12, #27, and #28 (Ppc-1). We also examined 23 natural compounds derived from other microorganisms and plants, and found 2 more compounds with oxygen consumption-stimulating activity. Of the 6 selected compounds, five significantly inhibited the rate of ‘state 3’ (Fig. 1B). Only Ppc-1 lacked inhibition of the rate of ‘state 3’, therefore, we selected this compound for further analysis.

**Figure 2:**
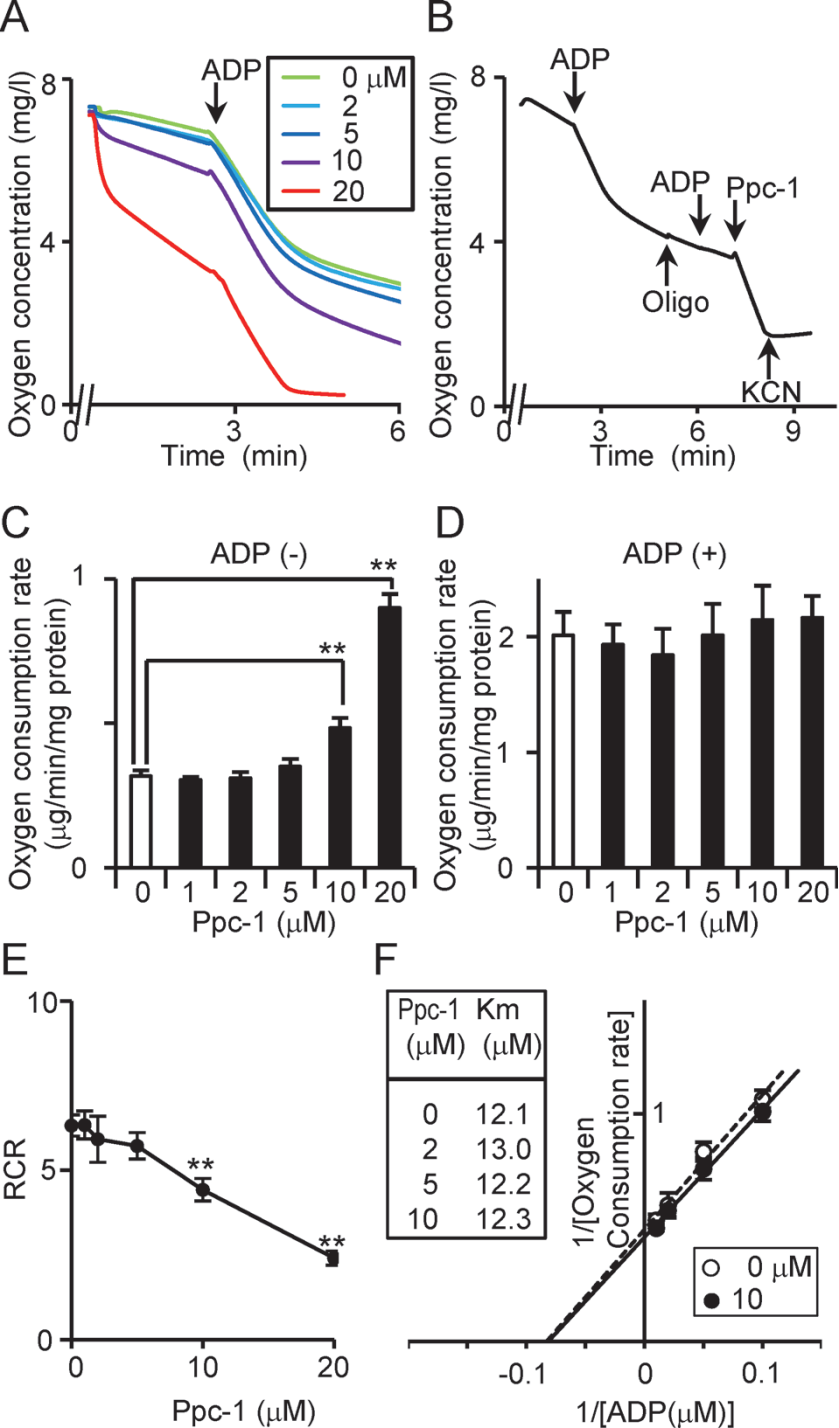
Oxygen consumption induced by Ppc-1. (A) Enhanced oxygen consumption was induced by Ppc-1 in a dose-dependent manner. Mitochondria-enriched fraction (0.1 mg protein) in assay buffer was pre-incubated at 30°C for 0.5 min, and the indicated amounts of Ppc-1 were added to the mixture at time point 0 min. Recording of oxygen level was started at 0.5 min after the addition of the compound, and then an aliquot of ADP was added to a final concentration of 400 μM to induce ‘state 3’. (B) Effects of oligomycin A (0.75 μM) and KCN (1 mM) on the oxygen consumption were examined. (C, D) The oxygen consumption rate in the absence (C) or presence (D) of ADP was calculated. Ppc-1 showed a significant increase in oxygen consumption at concentrations above 10 μM, but no effect on ‘state 3’ at any dose tested. **p < 0.01. (E) The RCR for ADP was calculated, confirming that Ppc-1 acts as a mitochondrial uncoupler. (F) Effect of Ppc-1 on the oxygen consumption rate was examined in assay buffer containing various concentrations of ADP. A representative reciprocal plot is shown, and the *K*m values for ADP are inserted.

Ppc-1 enhanced oxygen consumption rate in a dose-dependent manner (Fig. 2A). The effects of oligomycin A and KCN were used to confirm the effects of Ppc-1 on respiration; while the cytochrome c oxidase inhibitor, KCN, completely suppressed the effect of Ppc-1 on oxygen consumption rate, the ATP synthase inhibitor, oligomycin A, had no effect (Fig. 2B). Whereas Ppc-1 concentrations above 10 μM significantly enhanced oxygen consumption rate (Fig. 2C), no significant inhibition of ‘state 3’ was observed, even at such high concentration (Fig. 2D). The respiratory control ratio (RCR) for ADP suggests that Ppc-1 acts as a mitochondrial uncoupler (Fig. 2E). The apparent *K*m for ADP was 10–15 μM in the presence of varying concentrations of Ppc-1 (Fig. 2F), which were consistent with previous reports [25,26]. This confirms that Ppc-1 does not affect the function of ATP synthase.

### Ppc-1 suppresses weight gain in animals

**Figure 3:**
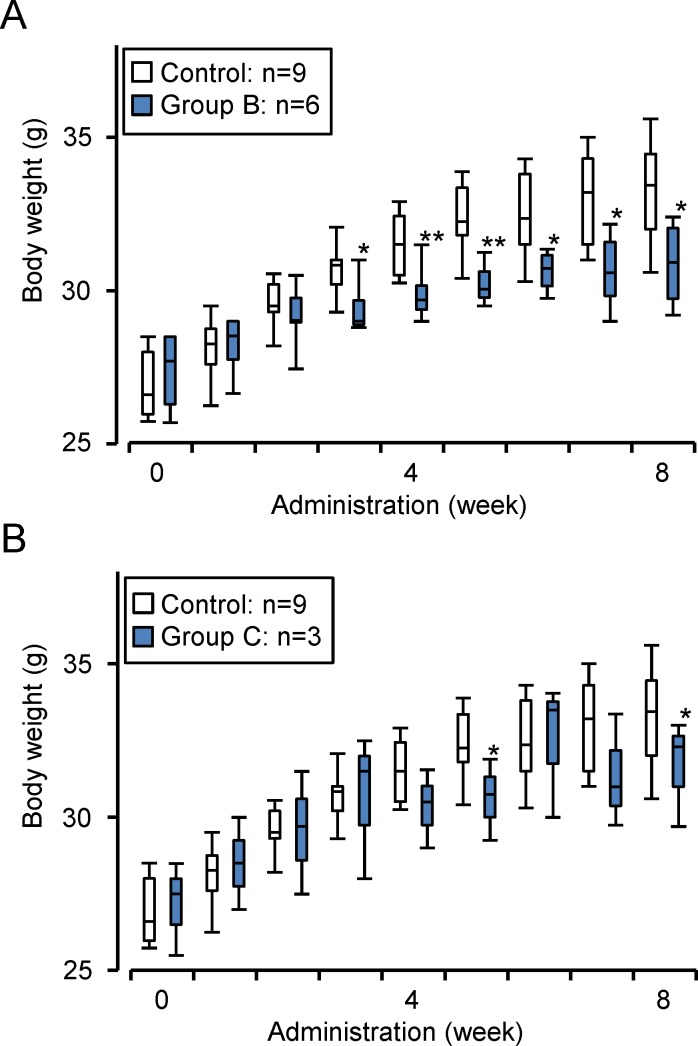
Suppression of weight gain by Ppc-1. Ppc-1 was injected once a week into the peritoneal cavity of mice for 8 weeks. Doses of Ppc-1 for groups B and C were 0.8 (A) and 4 (B) mg/week/kg, respectively. Results are expressed using box plots. **p < 0.01, *p < 0.05.

Uncoupling agents reduce the mitochondrial proton gradient, decreasing the efficiency of ATP production. It is well known that chronic administration of uncoupling agents in animals causes significant weight loss. Therefore, we examined the effect of Ppc-1 administration on weight and fat content in mice. ICR mice were divided into 5 groups (3–9 animals in each group), and Ppc-1 dissolved in phosphate buffered saline (PBS) was injected into the peritoneal cavity of each mouse once a week for 8 weeks. The Ppc-1 doses used were 0 (control), 0.16 (group A), 0.8 (group B), 4 (group C), and 10 mg/week/kg (group D). Since mice feed in the dark, body weight was measured every 2 days just before the lights were turned off. The most significant suppression of weight gain was observed in the group B mice (Fig. 3A), 3 weeks after the start of Ppc-1 administration, and this suppression was maintained throughout the experiment. Mean weights for the control and group B mice were respectively 32.3 g and 30.1 g after 5 weeks, and respectively 33.2 g and 30.6 g after 7 weeks, indicating 7~10% weight loss in Ppc-1 treated animals. Significant weight loss with Ppc-1 was also observed in the group C mice (Fig. 3B), although the effect was less pronounced. The weight differences between treated and the control animals were sustained, even after Ppc-1 administration was discontinued. On the other hand, weight differences were not significant between the control and group A or group D mice (S1 Fig.).

### Increase in serum fatty acid levels by Ppc-1 treatment

**Figure 4:**
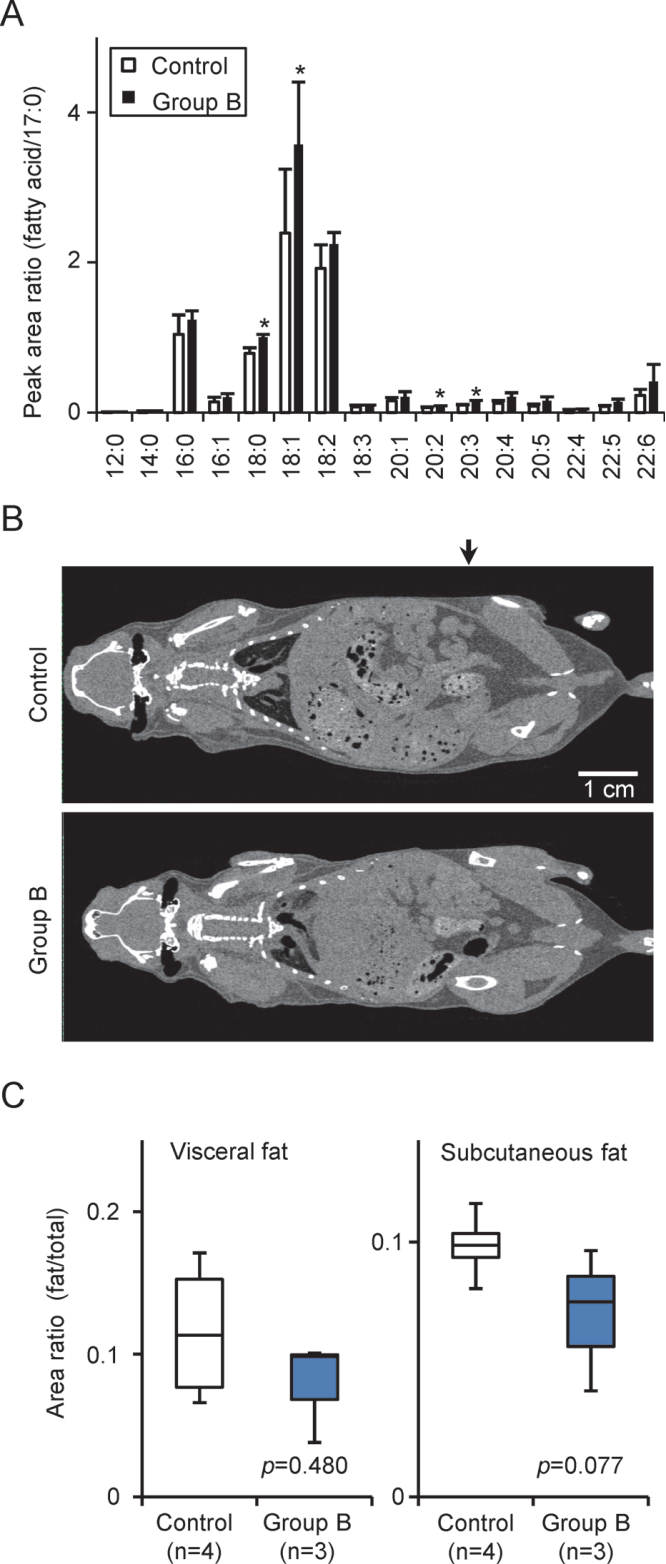
Fat analysis of Ppc-1-treated mice. (A) Free fatty acids in the serum of mice administered Ppc-1 at a dose of 0.8 mg/week/kg for 8 weeks were analyzed by HPLC-MS/MS system A. (B) Whole body fat was examined by a CT scanner, and representative vertical section images are shown. Fat contents were quantified from the cross section images, and the results are expressed using box plots. The position analyzed is denoted by an arrow. *p < 0.05.

We next compared components of serum from the control and group B mice. Three mice from each group were selected at random, and serum was collected from each animal on the 5th day after the final administration of Ppc-1. As shown in Table 1 and S1 Table, triacylglycerol and free fatty acids were significantly increased in Ppc-1-treated animals, suggesting enhanced lipid metabolism in response to the compound. Analysis of serum by mass spectrometry (MS) also revealed enhancement of specific fatty acids in response to Ppc-1, particularly stearic acid (18:0) and oleic acid (18:1) (Fig. 4A). These results were confirmed by computed tomography (CT) scanning (Fig. 4B and S2 Fig.) where the subcutaneous and visceral fat levels in the group B mice were respectively 7.2 ± 2.8% and 7.9 ± 3.6%, lower than the values measured in control mice of 9.9 ± 1.4% and 11.6 ± 5.1% (mean ± SD, n = 3). Furthermore, in contrast to the control animals, group B mice showed no ectopic fat accumulation.

**Table 1:** Profiling of Serum Factors.

	Triglyceride (mg/l)		Free fatty acid (μEQ/l)		Adiponectin (mg/l)		BUN (mg/l)		AST (IU/l)		ALT (IU/l)	
	Mean	SD	Mean	SD	Mean	SD	Mean	SD	Mean	SD	Mean	SD
Control	733	61	508	117	17.1	3.4	229	71	54.7	15.4	6.3	3.5
Group A	740	85	582	83	17.0	2.5	204	30	72.3	17.1	6.4	3.6
Group B	943*	102	666*	84	16.4	4.0	229	18	48.0	17.8	3.7	0.6
Group C	653	253	893*	187	18.1	3.9	230	24	57.0	6.1	4.3	0.6
Group D	718	299	990	444	14.6	1.1	260	51	83.5	44.5	12.0	7.1

*: *p*<0.05.

**Figure 5:**
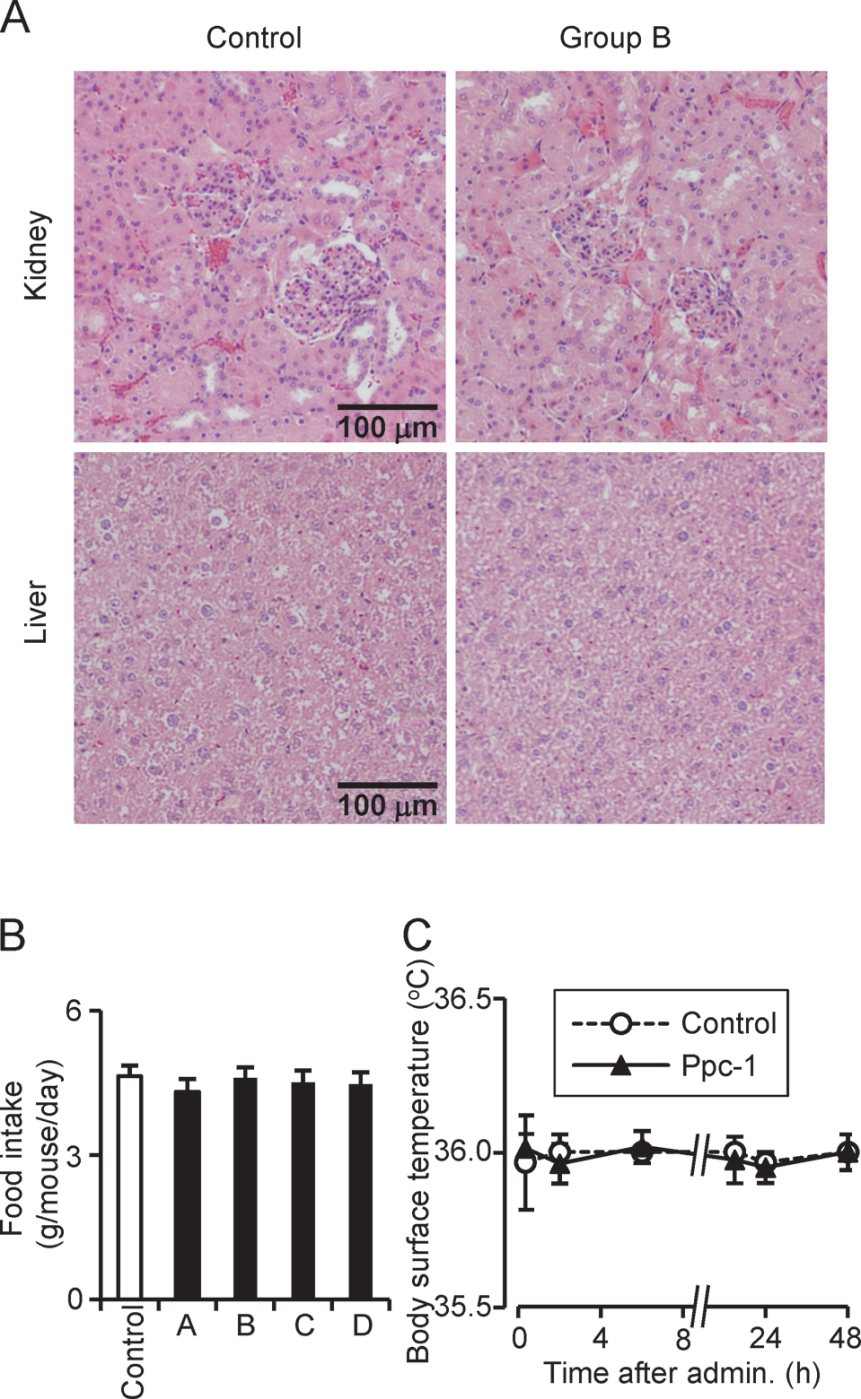
Examination of Ppc-1-treated mice. (A) Cross sections were prepared of fixed kidney and liver tissues from mice in the control and Ppc-1 treated (0.8 mg/week/kg) groups and stained with hematoxylin-eosin. Representative images are shown; no significant differences were detected between the two groups. (B) Daily food intake in 4 groups was recorded throughout the experiments. (C) Body surface temperature was monitored with an infrared thermometer following a single injection of Ppc-1 (0.8 mg/kg).

After serum collection, three mice from each group were euthanized and examined for tissue damage to liver and kidney. Tissue images are shown in Fig. 5A. Both liver and kidney images appeared normal, and no lesional changes were detected, including ectopic accumulation of fat. The remaining control and group B mice were housed under normal conditions and examined periodically until they died of natural causes. The levels of daily food intake were comparable among the control and Ppc-1 treatment groups (Fig. 5B). No abnormal behavior, evidence of tumor formation, or differences in life span was observed between the animals in the control and the four Ppc-1 treatment groups.

### Tissue distribution of Ppc-1

**Figure 6:**
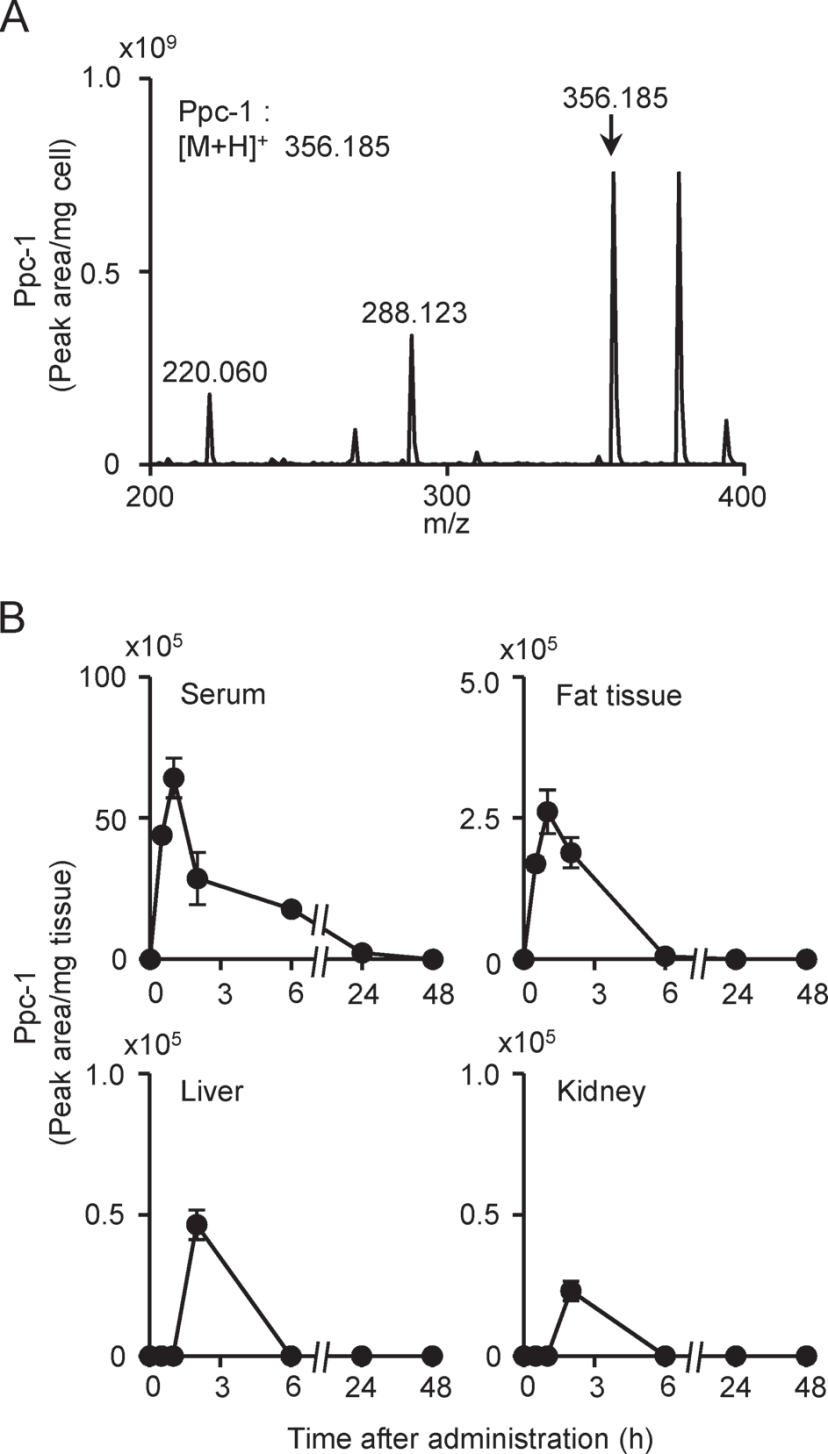
Detection of Ppc-1 in cells and tissues. (A) RPE cells were treated with 10 μM Ppc-1 for 12 h, and then lipid fraction was prepared from cells, and analyzed on HPLC-MS/MS system B. The positions of Ppc-1 ([M+H]^+^ = 356.19 *m/z*) and its derivatives are indicated. (B) Sera and tissues were collected from mice at the time points indicated after a single shot of Ppc-1 (0.8 mg/kg), and the lipid fraction was prepared and analyzed similarly. The peak areas were calculated, and the results are expressed as mean and SD.

Although Ppc-1 functions as an uncoupler and enhances oxygen consumption in mitochondria (Fig. 2C), no increase in body temperature was detected in mice treated with the reagent (Fig. 5C). However, Ppc-1 caused growth inhibition when added to mammalian cells at concentrations above 10 μM [19] (S3 Fig.). Therefore, we determined Ppc-1 concentrations within various tissues in mice treated with a single injection of Ppc-1 (0.8 mg/kg), collecting liver, kidney, visceral fat, and serum from the animals at different times after injection. Lipids were extracted from tissues with chloroform/methanol, and analyzed by LC-MS. The lipid fraction from RPE cells treated with 10 μM Ppc-1 for 12 h was also extracted and analyzed by LC-MS. A mass spectrum of Ppc-1 in RPE cells is shown in Fig. 6A; the major peak at *m/z* 356.19 was identified as intact Ppc-1 [M+H]^+^, and other peaks (e.g. *m/z* 288.12) were recognized as derivatives of Ppc-1. Ppc-1 concentrations were low in the sera and tissues derived from mice administered the compound. At 1 h after animal injection, Ppc-1 was detected in serum and fat tissue, at concentrations (68 and 2.6 x 10^5^ peak area/mg, respectively) which were less than 1% of that seen in cultured cells treated with 10 μM Ppc-1 (0.82 x 10^9^ peak area/mg) (Fig. 6B). The signal intensity of Ppc-1 in tissues declined gradually to basal levels, 24 to 48 h after injection. The Ppc-1 concentrations in liver, kidney, and subcutaneous fat were extremely low throughout the time points examined.

### Ppc-1 induces fatty acid release from adipocytes

**Figure 7:**
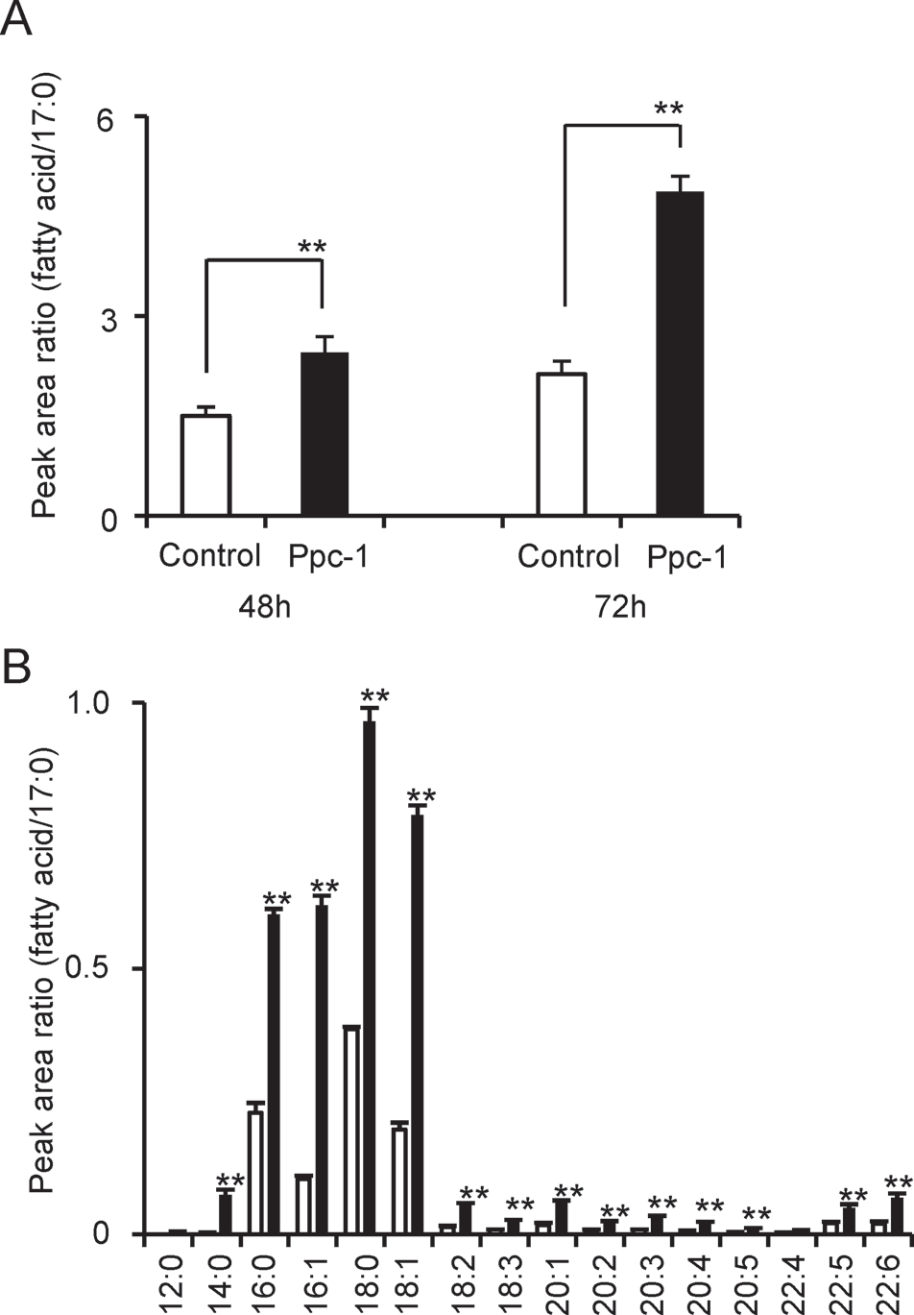
Release of fatty acids from 3T3-L1 cells by Ppc-1. Lipid extracts in the culture supernatants of differentiated 3T3-L1 cells treated with 10 μM Ppc-1 for 48 h or 72 h were analyzed by HPLC-MS/MS system. A. A representative analysis of total fatty acids (A), and fatty acid components (B) (72 h only) is shown. The peak areas were calculated, and results are expressed as mean and SD. **p < 0.01.

In an effort to understand why serum fatty acid levels are elevated in Ppc-1-treated mice (Table 1), the effects of the compound on adipocytes were examined using differentiated 3T3-L1 cells. Treatment of 3T3-L1 adipocytes with 10 μM Ppc-1 for 2–3 days led to significantly increased levels of free fatty acids in the culture medium, suggesting that Ppc-1 stimulates fatty acid release from adipocytes (Fig. 7). Therefore, it is possible that in animals, Ppc-1 entering the bloodstream from the peritoneal cavity stimulates adipocytes directly, promoting their release of fatty acids.

## Discussion

### Identification of Ppc-1 as a novel mitochondrial uncoupler

Small molecules that inhibit mitochondrial respiration have been indispensable for investigating mitochondrial function and regulation, and new modulators offer possibilities for uncovering mechanisms which are as yet undiscovered. In our screen of 32 natural compounds, Ppc-1 was especially interesting as a compound with oxygen consumption-stimulating activity but without inhibitory effect on ‘state 3’. ‘State 3’ is induced when both respiratory substrates and ADP are supplied to mitochondria. The electron transfer system and ATP synthase are strictly regulated, through molecular interactions and intra-mitochondrial signaling systems which under various conditions can be labile, reducing ATP synthesis and producing excess reactive oxygen species [27–30]. Ppc-1 at >98% purity enhances oxygen consumption rate in a dose-dependent manner (Fig. 2E), showing linear correlation with the RCR for ADP (Fig. 2E). However, Ppc-1 does not serve as a respiration substrate itself, and its oxygen consumption kinetics show that it does not affect ATP synthase (Fig. 2F). These results suggest that Ppc-1 is a novel mitochondrial uncoupler.

### Ppc-1 induces weight loss

We evaluated the effects of Ppc-1 on weight and fat content by administering it directly to mice. We found that Ppc-1 significantly suppresses weight gain in mice. It is noteworthy that this effect of Ppc-1 is observed at 21°C, a typical vivarium temperature that is below thermoneutrality [31]. Ppc-1 also leads to higher serum amounts of fatty acids and triacylglycerols, with lower percentages of subcutaneous and visceral fat compared to control mice. However, no other serum parameters show significant differences between Ppc-1-treated and control mice. The serum levels of ALT, BUN and adiponectin are all normal, and we observed no abnormalities in liver and kidney tissues. We initially guessed that Ppc-1 might induce adverse effects such as growth inhibition, since Ppc-1 inhibited growth of cultured cells [19] (S3 Fig.). However, we found that Ppc-1 concentrations were very low in serum and in all tissues tested in mice that were administered the compound (Fig. 7). Furthermore, no ectopic fat was detected in Ppc-1-treated mice. Thus, we speculate that Ppc-1 functions as an uncoupler in adipocytes and other cells in Ppc-1-treated animals, reducing the efficiency of mitochondrial ATP production. No excess heat production was observed, possibly because the Ppc-1 absorption and uncoupling reaction proceeded slowly over the course of serial administration. Low concentrations of Ppc-1 in the liver and kidney suggest a rapid degradation or modification by metabolic pathways. On the other hand, it is interesting that Ppc-1 stimulates adipocyte-like 3T3-L1 cells to release fatty acids into the culture medium (Fig. 7). Although the mechanism underlying the fatty acid release is under investigation, these findings are likely to be closely related to the increased serum fatty acids observed in mice treated with Ppc-1.

Obesity is a complex multifactorial disease and is a risk factor for other chronic diseases, including type 2 diabetes and cardiovascular disease. Understanding the metabolic factors and mitochondria dysfunction associated with obesity and weight loss success is important for developing appropriate prevention and treatment strategies [32]. Drugs for weight loss have been sought by many studies. DNP is reported to cause rapid weight loss, but leads to an unacceptably high number of adverse side effects [33–35]. Amphetamine derivatives have been used as appetite suppressants, but induce side effects that include valvular heart disease and lung hypertension. A new centrally active appetite suppressant and a lipase inhibitor are currently being used as diet pills, but have equally unpleasant side effects. A small molecule, AdipoRon, which binds to adiponectin receptors, is currently being developed for the treatment of type 2 diabetes and obesity [36]. Adiponectin is an anti-diabetic and anti-atherogenic adipokine that binds to adiponectin receptors AdipoR1 and AdipoR2, and exerts anti-diabetic effects *via* the activation of the AMPK and PPAR-α pathways. Since AdipoRon is orally active, a therapeutic approach using AdipoR agonists is promising for the treatment of diabetes and other obesity-related diseases. Alternatively, it has been reported that DNP-methyl ester safely decreases hypertriglyceridemia and insulin resistance without systemic toxicity in rats fed a high fat diet [37]. DNP-methyl ester is a liver-targeted derivative of DNP, demonstrating the potential feasibility of dissociating the toxicity of DNP from its efficacy by altering its pharmacokinetics. DNP-methyl ester is a promising new drug candidate for the prevention of nonalcoholic fatty acid liver and type 2 diabetes. In contrast to these compounds, the mechanism of action for Ppc-1 is obscure, and the effective dose range that induces significant weight loss is quite narrow; weight loss occurs at an intermediate dose of 0.8 mg/week/kg (group B mice), with less weight loss at higher doses of 4 mg/week/kg and 10 mg/week/kg, and no weight loss at a lower dose (0.16 mg/week/kg). Thus, it will be necessary to evaluate the effects of Ppc-1 in mice fed high-fat diets and other fatty liver models, as well as optimize the form of administration including oral and percutaneous routes.

### Action mechanism of Ppc-1

The molecular targets of Ppc-1 and its uncoupling mechanisms remain under investigation. Preliminary results indicate that uncoupling by Ppc-1 does not depend on the uncoupling proteins, UCP1 and UCP2. Furthermore, the uncoupling effect of Ppc-1 must be independent of ATP production, including respiratory complex V, because there is no inhibitory effect on ‘state 3’. We considered whether Ppc-1 stimulates the formation of the permeability transition pore (PTP) [38], resulting in proton inflow to the matrix and uncoupling. The PTP modulates increased mitochondrial inner membrane permeability to ions and solutes with molecular masses up to about 1,500 Da, which causes matrix swelling. Thus, the PTP is central to vital mitochondrial functions and can play a lethal role under many pathophysiological conditions. However, Ppc-1 does not stimulate mitochondrial swelling nor apoptosis; therefore, the PTP is not likely to be the target of Ppc-1. Further studies are needed to address the molecular mechanism of Ppc-1.

## Conclusions

Ppc-1 derived from slime molds was isolated from a library of small molecules as a novel agent which stimulates oxygen consumption in mitochondria with no inhibitory effects on ATP production. Direct administration of Ppc-1 to mice suppresses weight gain without abnormal changes in liver and kidney tissues, and no detectable tumor formation. The levels of free fatty acids are significantly elevated in the serum of mice treated with Ppc-1, while body fat content is relatively low. Ppc-1 stimulates cultured adipocytes to release fatty acids into the medium, which might be related to the rise in fatty acid levels in the serum of Ppc-1-treated mice. Our results suggest that Ppc-1 is a unique uncoupling agent applicable as a molecular probe for mitochondria research, and likely to aid the development of new drugs for the treatment of obesity.

## Methods

### Animal experiments

All experiments were carried out with the approval of the Fukushima Medical University Animal Studies Committee. Female ICR mice were obtained from CLEA Japan (Tokyo, Japan), and housed at 21°C with a 12:12 h light:dark cycle with free access to water and a commercial diet. Ppc-1 dissolved in dimethyl sulfoxide was diluted with PBS and injected once a week into the peritoneal cavity for 8 weeks. Injections started when body weight was over 25 g, and doses of Ppc-1 varied between 0 and 10 mg/kg. Body weight and food intake were measured every 2 days just prior to lights out. Serum was collected from each animal on day 5 after the final administration of Ppc-1, and serum components including the albumin:globulin ratio, triglycerides, free fatty acids, creatinine, γ-GTP, aspartate aminotransferase (AST) and urea-N (BUN) were measured. Three mice were selected at random from each group, and euthanized for body fat analysis and tissue examination. Body fat was analyzed by LaTheta LCT-200 CT (Hitachi-Aloka, Tokyo). Livers and kidneys were fixed in paraformaldehyde and 6-μm-sections were prepared. For detection of Ppc-1 in various tissues, liver, kidney, visceral fat, and serum were collected from mice treated with 0.8 mg/kg Ppc-1 for various time periods. Body surface temperature was monitored with an infrared thermometer.

### Chemicals

The compounds screened in this study are listed in Fig. 1A; all were derived from *Dictyostelium* cellular slime molds. These compounds were synthesized and purified as described [8,9,20–24], and the structures and purities were confirmed by ^1^H and ^13^C NMR spectroscopy and high resolution MS. The purities of all compounds were greater than 98%. Oligomycin A, palmitoyl-L-carnitine, and fat-free bovine serum albumin (BSA) were purchased from Sigma-Aldrich.

### Preparation of mitochondria-enriched fractions

Mitochondria were isolated from mouse liver (ICR; 7–10 week old females) by differential centrifugation as described previously [28]. Cells were homogenized in a Potter glass homogenizer with H-Buffer (250 mM sucrose, 10 mM Tris-HCl, pH 7.4), and centrifuged at 800xg for 1 min at 4°C. The supernatant was centrifuged at 6,000xg for 5 min, and the resulting pellet, the crude mitochondrial fraction, was suspended in H-Buffer. The suspension was layered over a discontinuous sucrose gradient consisting of 1.0 M and 1.5 M sucrose in Tris buffer (10 mM Tris-HCl, pH 7.4), and centrifuged for 20 min at 26,000xg at 4°C. The interface was collected in Tris buffer and centrifuged at 6,000xg for 5 min. The resulting pellets were suspended in Tris buffer (3 mg protein/ml) and used for experiments after confirming the presence of the mitochondrial marker cytochrome c.

### Oxygen consumption analysis

Mitochondrial oxygen consumption was measured using a Clark-type oxygen electrode (Strathkelvin Instruments, North Lanarkshire, Scotland) as described [28–30,39]. Prior to the assay, an aliquot of mitochondrial-enriched fraction (0.1 mg protein) was incubated at 30°C in oxygen measurement buffer (225 mM mannitol, 75 mM sucrose, 10 mM KCl, 0.1 mM EDTA, 3 mM phosphate, 100 μM palmitoyl-L-carnitine, and 20 mM Tris-HCl, pH 7.4) for 0.5 min. A test compound was added to the mixture at time = 0. Recording of oxygen level was started 30 s after the addition of the compound, and then an aliquot of ADP was added to a final concentration of 400 μM to induce ‘state 3’. Oligomycin A and KCN were used as respiratory system inhibitors.

### Cell culture

RPE cells were grown and maintained at 37°C under 5% CO_2_ and 95% air in Dulbecco’s modified Eagle’s medium-low glucose (DMEM-low glucose, 5 mM glucose) supplemented with 10% (v/v) heat-inactivated fetal bovine serum (FBS) in a humidified incubator. 3T3-L1 fibroblasts were maintained in DMEM-high glucose (25 mM) containing 10% FBS. Post-confluent cells were differentiated into adipocytes by culturing for 3 days in medium containing 1 μg/ml insulin, 0.1 μg/ml dexamethasone, and 112 μg/ml isobutylmethylxanthine [40,41]. Then the differentiation medium was replaced with DMEM, and the cells were cultured further for 10–14 days with medium changes every 4 days. Approximately 90% of cells showed the presence of large lipid droplets. Prior to the addition of Ppc-1, cells were maintained in a serum-free, BSA-containing (2%, w/v) medium for 1 day [41].

### Mass spectrometry

Fatty acids and Ppc-1 in sera, tissues, and cells were analyzed by C18 reverse-phase liquid chromatography coupled to high resolution MS as reported [42,43]. Briefly, lipids and organic compounds were extracted by the method of Bligh and Dyer [44]. The free fatty acid compositions were analyzed on high performance liquid chromatography (HPLC)-MS/MS system A (HPLC, UltiMate 3000; MS, TSQ Vantage, Thermo Scientific) controlled by XCALIBUR (version 2.2, Thermo Scientific). To assess relative concentrations, the peak areas obtained from the MRM chromatogram for each fatty acid were normalized to the peak area of C17:0 margaric acid added as an internal standard. For the detection of Ppc-1, the extracts were analyzed on HPLC-MS/MS system B (HPLC, UltiMate 3000; MS, Orbitrap, Thermo Scientific) controlled by XCALIBUR. The *m/z* list of Ppc-1 was obtained from Metworks (version 1.3, Rococo Co., Ltd, Osaka, Japan) software for the prediction of *m/z* of drugs and their derivatives. The peak values of Ppc-1 were calculated from mass spectra obtained by positive acquisition polarity in a mass spectrometer.

### Statistical analysis

Data were assessed using ANOVA with Tukey-Kramer post-hoc comparisons, and values are expressed as mean and SD unless otherwise indicated. The weights and fat levels of mice in the control and treated groups were evaluated by the Mann-Whitney test. Two-tailed values with *p*<0.05 were considered statistically significant.

## Supporting Information

S1 Fig Weight changes following Ppc-1 treatment. Ppc-1 was injected once a week into the peritoneal cavity for 8 weeks. Doses of Ppc-1 for group A and group D were 0.16 (A) and 10 (B) mg/week/kg. No significant weight loss was observed in these groups.
(TIF)

S2 Fig Body fat levels were examined using a CT scanner; representative cross section images of control and group B mice are shown (n = 3, each group). The position analyzed is denoted by an arrow in Fig. 4B.
(TIF)

S3 Fig Effect of Ppc-1 on cell growth and ATP metabolism. (A) Cells were treated with the indicated doses of Ppc-1 for 48 h, and cell numbers were determined by counting. (B) ATP synthase activity was determined using the mitochondrial-enriched fraction. After 5 min incubation in oxygen measurement buffer containing the indicated amounts of Ppc-1, ADP was added to the reaction mixture to induce ‘state 3’. ATP contents were measured 1 min before and 1 min after the addition of ADP, and the ATP synthesis rates were calculated from the difference in the two values. (C) ATPase activity was determined using mitochondria-enriched fraction, and the enzyme activity was measured as reported [45].
(TIF)

S1 Table Body Fat and Profiling of Serum Factors. (DOCX)
