# Supplementary figures and images for: Weight Loss by Ppc-1, a Novel Small Molecule Mitochondrial Uncoupler Derived from Slime Mold
Source: PLoS One. 2015 Feb 10;10(2):e0117088. doi: 10.1371/journal.pone.0117088 (PMC4323345; doi:10.1371/journal.pone.0117088)

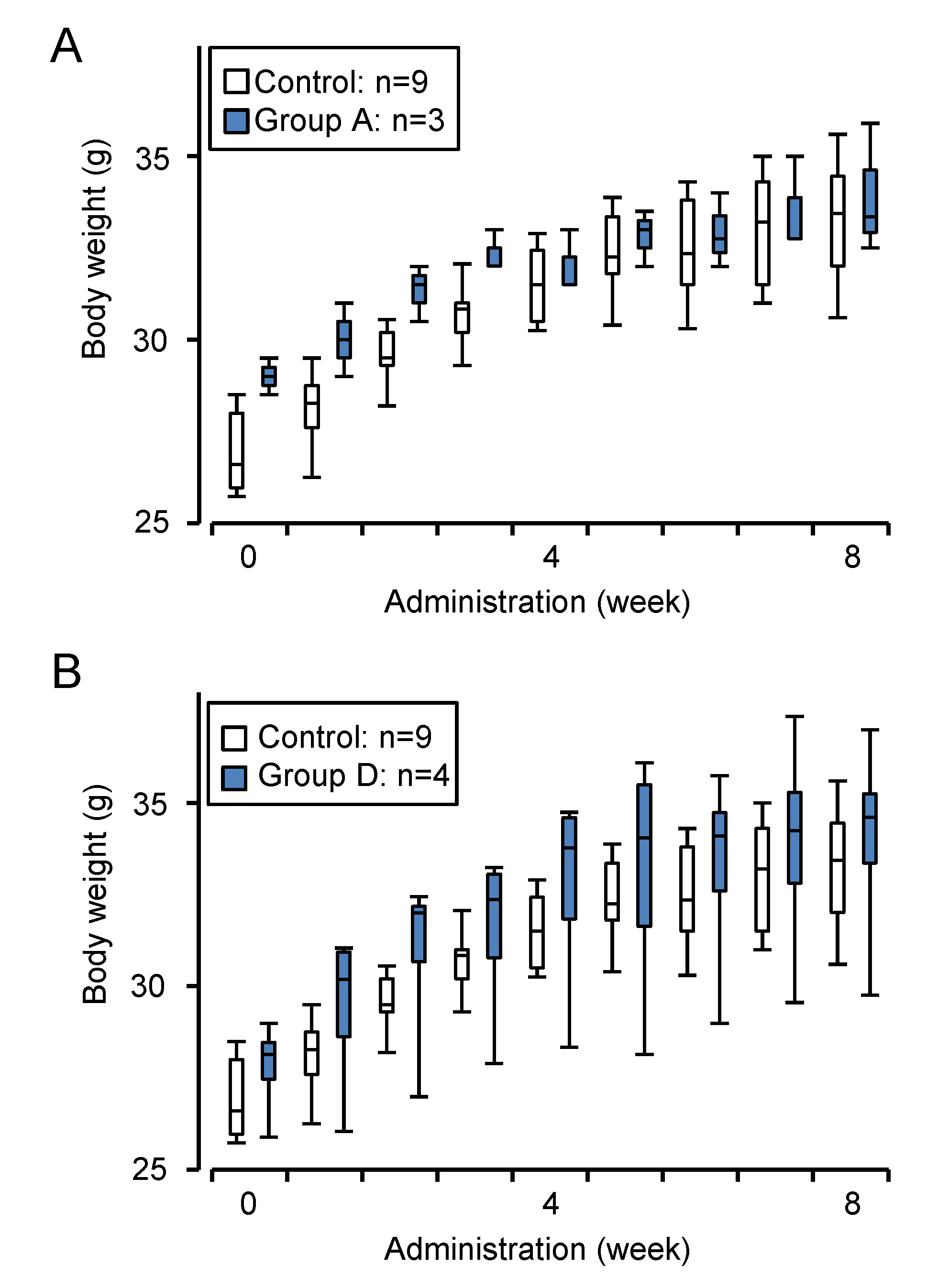

Supplement: S1 Fig — Ppc-1 was injected once a week into the peritoneal cavity for 8 weeks. Doses of Ppc-1 for group A and group D were 0.16 (A) and 10 (B) mg/week/kg. No significant weight loss was observed in these groups. (TIF) [file pone.0117088.s001.tif]

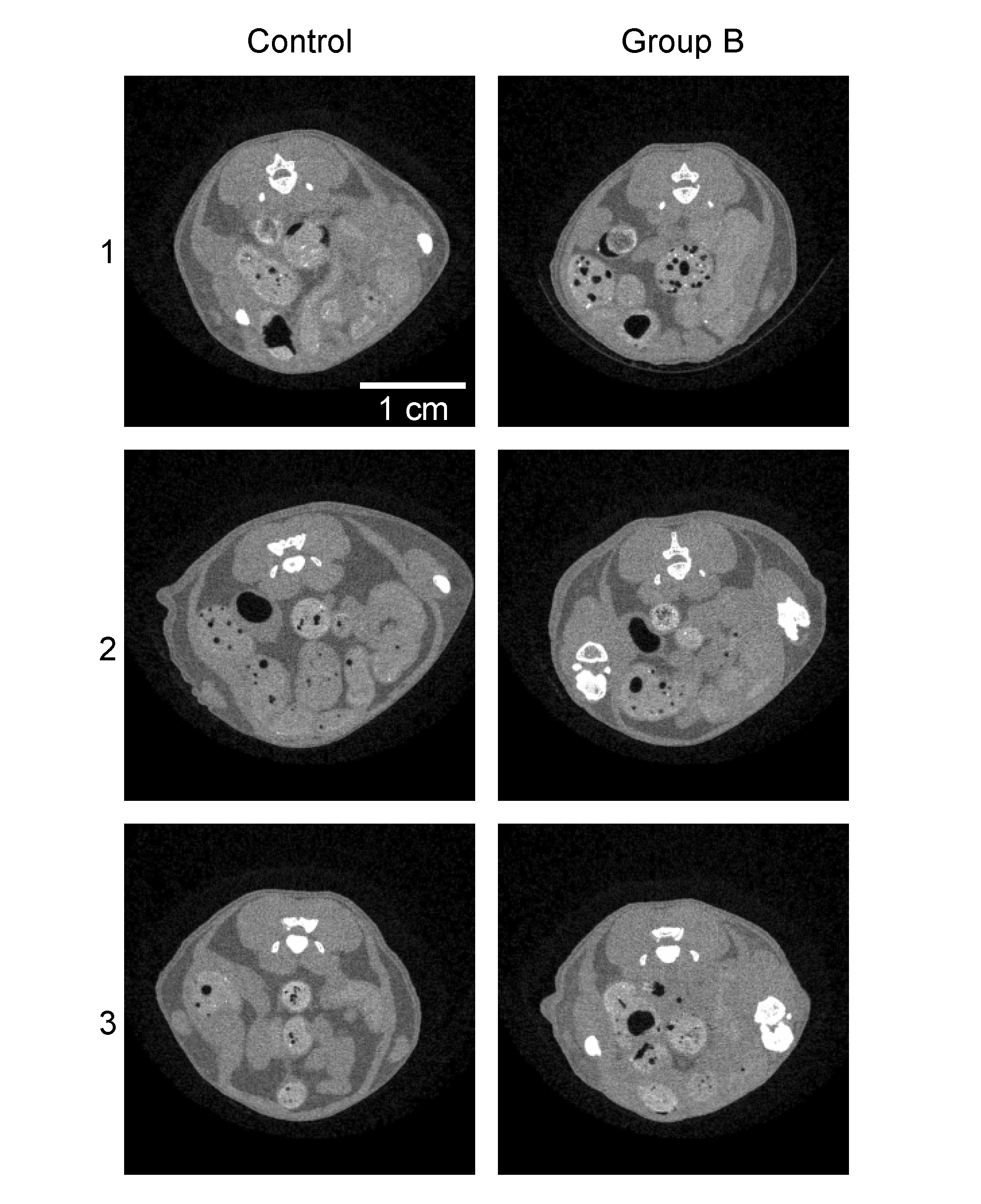

Supplement: S2 Fig — The position analyzed is denoted by an arrow in Fig. 4B. (TIF) [file pone.0117088.s002.tif]
