# Supplementary material for: Weight Loss by Ppc-1, a Novel Small Molecule Mitochondrial Uncoupler Derived from Slime Mold
Source: PLoS One. 2015 Feb 10;10(2):e0117088. doi: 10.1371/journal.pone.0117088 (PMC4323345; doi:10.1371/journal.pone.0117088)
Supplement: S1 Table — (DOCX) [file pone.0117088.s004.docx]

Supporting Information Table S1. Body Fat and Profiling of Serum Factors

|  | Percent body fat (%) | | Total cholesterol (mg/l) | | Phospholipid (mg/l) | | Albumin / Globulin | | Creatinine (mg/l) | | γ-GTP (IU/l) | |  | |  | |
| --- | --- | --- | --- | --- | --- | --- | --- | --- | --- | --- | --- | --- | --- | --- | --- | --- |
|  | Mean | SD | Mean | SD | Mean | SD | Mean | SD | Mean | SD | Mean |  |  |  |  |  |
| Control | 15.1 | 2.9 | 778 | 121 | 1,302 | 164 | 1.88 | 0.10 | 1.50 | 0.61 | <3 |  |  |  |  |  |
| Group A | 15.1 | 1.8 | 820 | 123 | 1,407 | 179 | 1.93 | 0.12 | 1.10 | 0.26 | <3 |  |  |  |  |  |
| Group B | 11.3 | 4.5 | 708 | 81 | 1,290 | 168 | 1.70 | 0.18 | 1.68 | 0.48 | <3 |  |  |  |  |  |
| Group C | 14.5 | 1.1 | 788 | 158 | 1,340 | 144 | 1.88 | 0.21 | 1.58 | 0.38 | <3 |  |  |  |  |  |
| Group D | 17.9 | 2.6 | 740 | 158 | 1,305 | 288 | 2.03 | 0.22 | 1.53 | 0.34 | <3 |  |  |  |  |  |
